# Emergency treatment of acute massive hemorrhage during transbronchial lung biopsy outside operating room: Case report

**DOI:** 10.1097/MD.0000000000050952

**Published:** 2026-10-09

**Authors:** Shuxian Ma, Sichen Cui, Lining Huang, Hongmei Lu

**Affiliations:** a Department of Anesthesiology, The Second Hospital of Hebei Medical University, Shijiazhuang, Hebei, China.

**Keywords:** case report, emergency, hemorrhage, outside operating room, transbronchial lung biopsy

## Abstract

**Rationale:**

Massive hemorrhage related to bronchoscopy procedures can occur suddenly, with rapid bleeding and difficulty in hemostasis, potentially leading to airway obstruction, asphyxiation, and even shock or death. The management of acute massive hemorrhage during transbronchial lung biopsy procedures is particularly critical.

**Patient concerns:**

This case report describes a patient with a complex condition who experienced massive bleeding during a fiberoptic bronchoscopy lung biopsy.

**Diagnoses:**

The patient had a complex medical history, including community-acquired pneumonia (common type), emphysema, pulmonary nodule, postradiation therapy for breast cancer, post-PCI for coronary atherosclerotic heart disease, grade 3 hypertension (extremely high risk), and atrial fibrillation (arrhythmia).

**Interventions:**

Preoperative bleeding risk assessment and preventive measures were performed. During the emergency, rescue procedures included airway control via large-diameter endotracheal intubation, body position adjustment, drainage and suction, systemic and local hemostasis, fluid therapy, maintenance of a normal internal environment, and stabilization of vital signs.

**Outcomes:**

The operation was successfully completed. After the operation, the patient improved with hemostatic treatment and care and was discharged smoothly.

**Lessons:**

Controlling the airway to ensure ventilation is crucial during massive bleeding related to bronchoscopy procedures. Intubating a large-diameter endotracheal tube into the main airway can improve ventilation and provide time and opportunity for subsequent hemostatic measures. The rescue process requires close cooperation among the medical team and the use of various treatment measures, including adjusting body position, drainage and suction, intubation, systemic and local hemostasis, fluid therapy, maintaining a normal internal environment, and stabilizing vital signs, to ensure the patient transitions from danger to safety. Additionally, preoperative identification of high-risk patients and preventive and emergency strategies are also of significant importance in preventing massive bleeding during bronchoscopic biopsy. However, this is a single-case experience, and its safety and general applicability require further validation through larger studies.

## 1. Introduction

Transbronchial lung biopsy (TBLB) under fiberoptic bronchoscopy is a common method for diagnosing pulmonary parenchymal lesions. The procedure is highly invasive, causing significant discomfort to the patient, and is typically performed under sedation or anesthesia.^[1]^ Complications related to TBLB include bleeding, respiratory depression, pneumothorax, etc., which have a high mortality rate once they occur.^[2]^ Massive bleeding related to bronchoscopy procedures is defined as acute massive bleeding of the lower respiratory tract caused by bronchoscopy procedures, a single episode of bleeding ≥ 100 ml, either diagnostic or therapeutic. Due to its sudden onset, rapid bleeding, and difficulty in stopping it, it can quickly lead to airway obstruction, causing asphyxiation or even shock and death.^[3]^ The management of acute massive bleeding during TBLB is particularly critical.

## 2. Case report

A patient, height 163 cm, weight 57 kg, was admitted with complaints of chest pain for 2 months, cough with wheezing for 20 days, and fever for 12 days. Chest CT results upon admission showed (Fig. 1) – Multiple patchy and lobar infiltrates in both lungs, particularly prominent in the left upper lobe, with local consolidation and bronchial air bronchogram observed, suggestive of inflammation; Multiple lymph nodes in the mediastinum, the largest with a short diameter of approximately 1.1 cm. Her previous medical history includes hypertension for 10 years, with a maximum blood pressure of 190/90 mm Hg (1 mm Hg = 0.133 kPa). Currently, no oral antihypertensive medication was being taken, but regular blood pressure monitoring showed normal readings. She underwent percutaneous coronary intervention for coronary artery disease 1.5 years ago, with a history of atrial fibrillation for 2 years, and was on long-term oral medications including “15 mg Rivaroxaban once daily, 100 mg Propafenone 3 times daily, and 20 mg Atorvastatin once daily.” She also uderwent breast cancer surgery 10 months ago, completed radiotherapy 8 months ago, and was on long-term oral medication “1 tablet of Exemestane once daily.” The patient has been asymptomatic for over 2 months, undergoing a chronic course. Upon arrival, the patient was treated with anti-infection therapy at another hospital over a span of 16 days, yet the treatment’s efficacy remains unverified. Based on her medical history, it is not ruleable out that the patient may have underlying conditions such as severe pneumonia, specific bacterial infection, radiation pneumonitis, or metastasis arising from breast cancer. Therefore, a plan was developed to undergo fiberoptic bronchoscopy, including airway lavage and lung biopsy, to confirm the diagnosis. Preoperative diagnoses were made: Pneumonia (community-acquired, common type); Emphysema; Pulmonary nodule; postradiation therapy for breast cancer; Post-PCI for coronary atherosclerotic heart disease; Hypertension with grade 3 (extremely high risk); Atrial fibrillation (arrhythmia). Preoperative 24-hour Holter showed sinus rhythm with an average heart rate of 78 beats per minute; routine coagulation function results showed prothrombin time (PT) 23.8 seconds; INR 2.11; fibrinogen (Fib) 7.39 g/L. Rivaroxaban was discontinued 24 hours before surgery. Because preoperative liver function tests showed elevated transaminases and the patient had long-term antibiotic use that may inhibit colonic anaerobes responsible for vitamin K production, vitamin K_1_ (10 mg, intramuscular) was administered to enhance hepatic synthesis of coagulation factors. This measure was not intended to reverse rivaroxaban activity.The patient’s coagulation function improved accordingly (PT 16 s, INR 1.43 on the morning of the procedure). Airway assessment: Mallampati grade II, no micrognathia, macroglossia, no sleep apnea, mouth opening > 3 fingerbreadths.

**Figure 1.**
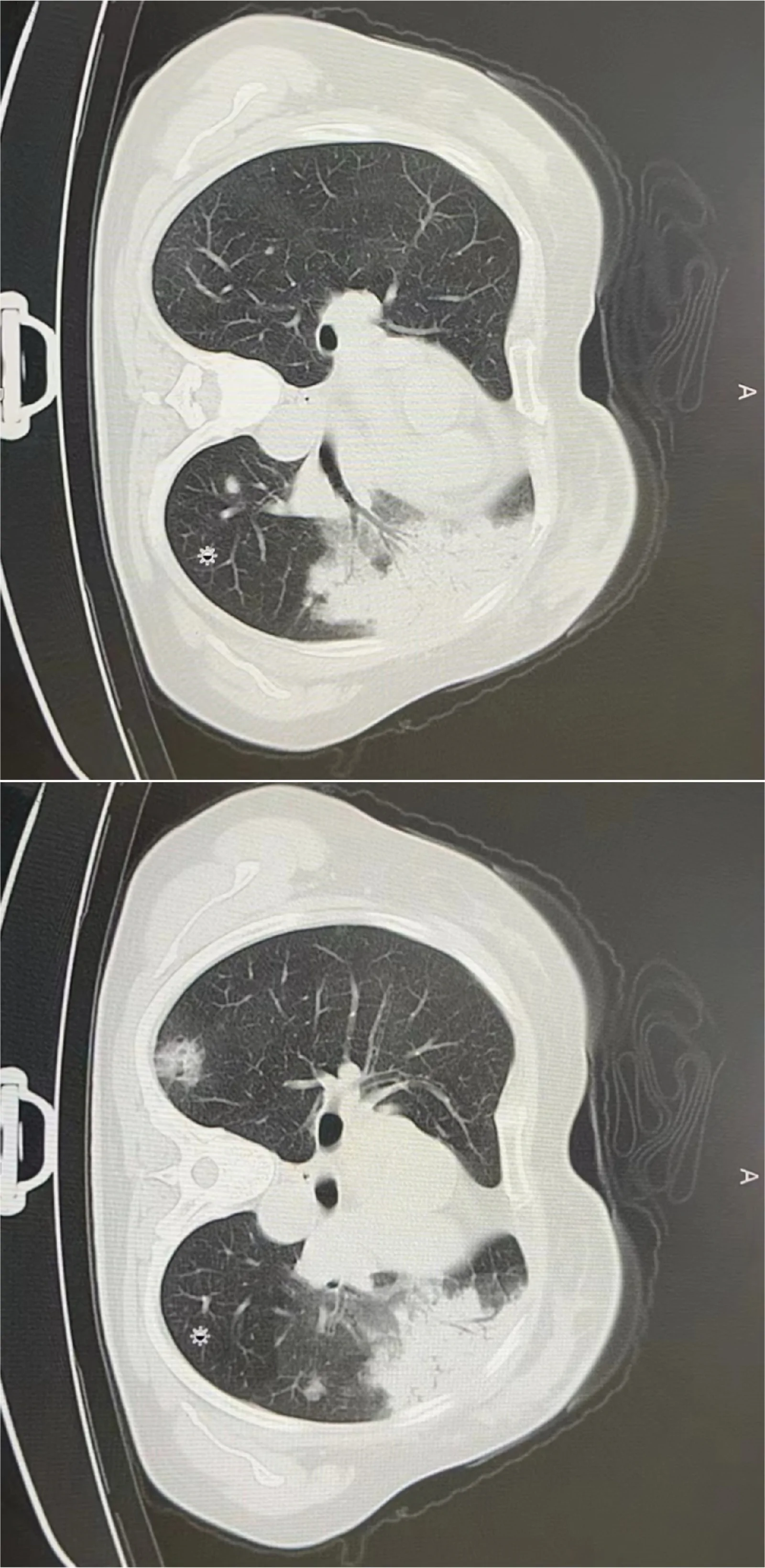
Chest CT results upon admission showed the following.

Preoperative fasting and water restriction were observed for 8 hours. Upon entering the bronchoscopy room, the patient was cooperative, with slight wheezing. The patient was placed in a supine position with a nasal cannula providing oxygen at 4 L/min. Vital signs were monitored: HR 86 beats/min, BP 137/73 mm Hg, RR 20 breaths/min, SpO_2_ 95%. Based on the surgical procedure and the anesthesiologist’s experience, the anesthesia method chosen was a combination of surface anesthesia and intravenous general anesthesia, maintaining moderate to deep sedation. After establishing a peripheral intravenous line, surface anesthesia was performed on the oropharynx and subglottic area. A mixture of 1% lidocaine and 2% tetracaine was prepared and sprayed in three sequential applications to the posterior half of the patient’s tongue, soft palate, pharyngeal wall, and larynx. Subsequently, 3 ml of the mixed solution was injected via the cricothyroid membrane, and the patient was instructed to cough several times to anesthetize the upper trachea, subglottis, and epiglottis. Anesthesia induction: an intravenous injection of oxycodone 5 mg was administered. Once effective, remimazolam 5 mg and etomidate 6 mg were administered intravenously. When the patient’s eyelash reflex disappeared, a fiberoptic bronchoscope was inserted. Anesthesia maintenance: intravenous infusion of remimazolam 0.3 to 0.5 mg/kg·h and cipepofol 0.5 mg/kg·h. The fiberoptic bronchoscope was inserted smoothly through the mouth, with good glottic closure and an unobstructed trachea. The mucosa appeared smooth under the scope, with no obvious secretions or neoplasms observed. A lavage in the upper lobe of the left lung was performed, and five brushing specimens were taken. During this period, the patient’s vital signs remained stable, spontaneous breathing continued, there was no severe coughing episodes, and no respiratory depression occurred.

A fiberoptic bronchoscopy-guided transbronchial lung biopsy (TBLB) was performed on the distal bronchus of the upper lobe of the left lung. Immediately after the biopsy, acute massive airway bleeding occurred. According to the Chinese Expert Consensus on the Prevention and Management of Bronchoscopic Procedure-Related Bleeding, a single bleeding volume ≥ 100 mL is defined as massive bleeding, the most severe complication of bronchoscopy. In this case, the total blood loss was approximately 300 mL, meeting this criterion. Within about 30 seconds of the biopsy, a large amount of bright-red blood rapidly flooded the airways, causing immediate airway obstruction and severe hypoxemia. Oxygen saturation fell from 97% to 70%, reaching a nadir of 50%, accompanied by bradycardia and hypotension (exact values not recorded). The patient was promptly placed in the left-lateral position, with the bleeding left lung in the dependent position. This can reduce blood flow into the contralateral (right) main bronchus, lower the risk of soiling the healthy right lung, and preserve gas‑exchange function of the unaffected lung during massive airway hemorrhage. And continuous suction through the bronchoscope removed about 150 mL of blood, but ventilation did not improve. Endotracheal intubation with an 8.0-mm tube was performed and connected to a ventilator. Because of persistent airway blockage, mechanical ventilation with 100% oxygen yielded a peak airway pressure > 35 cm H_2_O and tidal volume < 200 mL, with SpO_2_ remaining around 80%. Manual positive-pressure ventilation was initiated while suction continued through the bronchoscope. Over the next 2 hours, an additional ≈ 150 mL of blood and clots were removed, and SpO_2_ gradually recovered to 93% to 95%. These objective findings – a 300 mL blood loss within minutes, airway occlusion, and life-threatening desaturation – fulfill the national definition of bronchoscopy-related massive bleeding and demonstrate the clinical severity of this case. After subsequent hemostatic interventions, bleeding ceased and oxygenation stabilized. During the hemostatic management, intravenous hemocoagulase (1 kU) and pituitrin (5 U IV bolus followed by continuous infusion) were administered in accordance with the Chinese Expert Consensus on the Prevention and Management of Bronchoscopic Procedure-Related Bleeding, which recommends these agents as first-line options for acute procedure-related hemorrhage. Pituitrin acts by constricting bronchial arteries and typically takes effect within 3 to 5 minutes, while hemocoagulase accelerates fibrin formation and shortens bleeding time. Although evidence remains limited, these agents are widely adopted in China for emergency airway bleeding, as supported by the national consensus. The procedure was then completed successfully, lasting 3.5 hours in total. A large amount of bronchial tree-like clots were removed during the procedure (Figs. 2 and 3). The patient received 1000 ml of crystalloid fluid, 100 ml of sodium bicarbonate injection, and 2 U of suspended red blood cells. Postoperatively, the patient was intubated and transferred to the respiratory ICU. Upon admission to the ICU, the patient’s spontaneous breathing was observed, with a SpO_2_ 98%, HR 70 beats/min, BP 117/63 mm Hg. One hour later, the patient regained consciousness, was responsive. Bronchoscopy revealed no active bleeding. After suctioning, the endotracheal tube was removed. Four hours later, the patient returned to the general ward and received Level I care. From day 1 to day 3 postoperatively, the patient experienced minor hemoptysis. Intravenous administration of hemocoagulase (1 kU daily) and etamsylate continued postoperatively for hemostasis in accordance with the above consensus recommendations, and bleeding ceased completely by postoperative day 4. The patient’s condition was complex, and the lung biopsy pathology showed a small number of atypical cell clusters, requiring further puncture examination for a definitive diagnosis. Considering the patient’s history of bleeding and high risk of puncture, the patient and his family refused to carry out further examination and continued anti-inflammatory treatment. The patient’s cough and wheezing were relieved and he was discharged smoothly after his condition improved.

**Figure 2.**
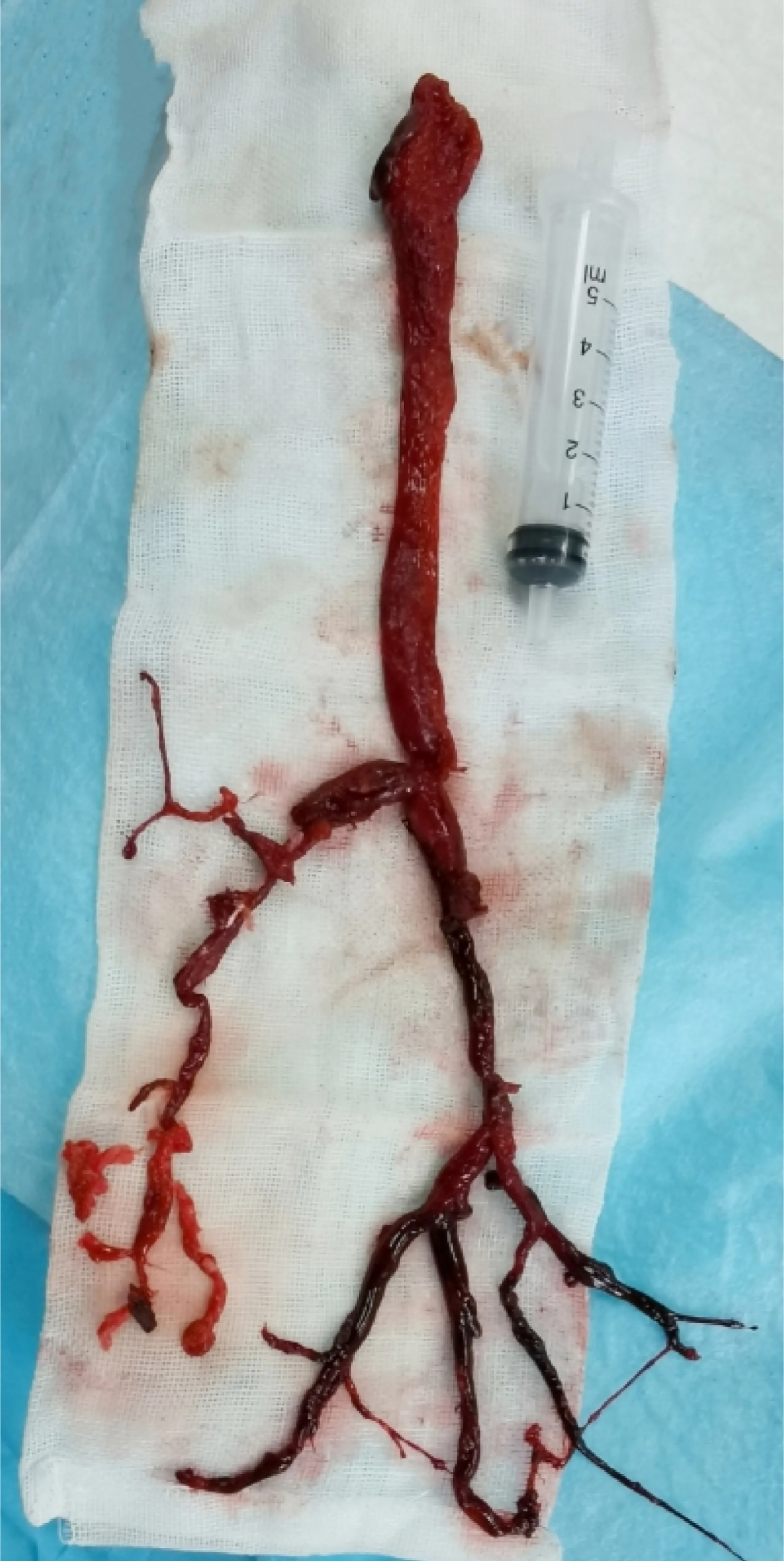
Gross specimen of the large amount of bronchial tree-like clots were removed during the bronchoscopic procedure.

**Figure 3.**
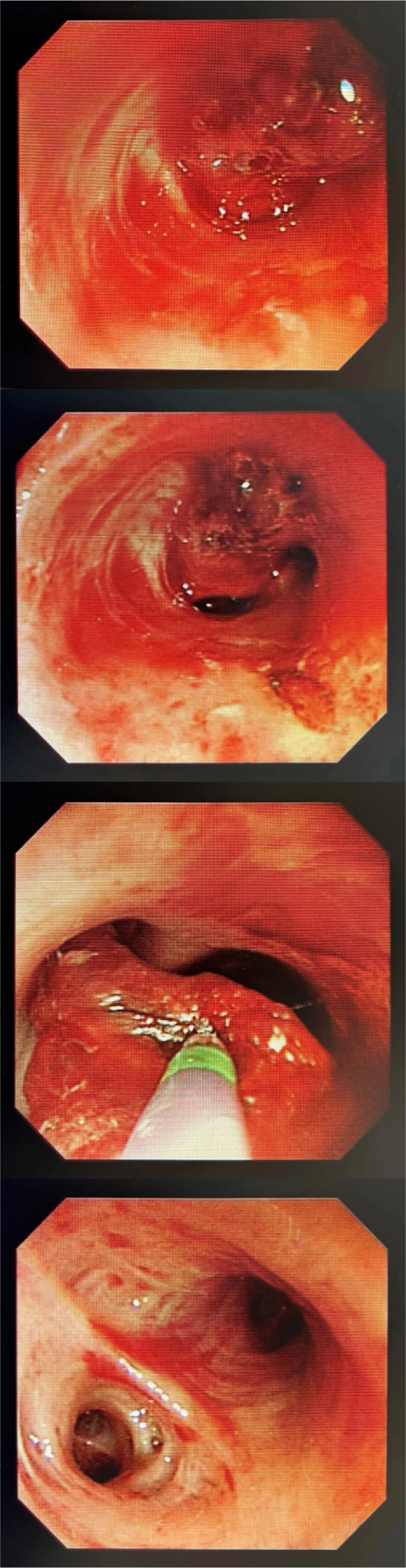
Intraoperative bronchoscopic surgery was ultimately completed successfully, lasting a total of 3.5 hours, with approximately 300 ml of blood loss.

## 3. Discussion

The TBLB procedure is currently the most commonly used method for the clinical diagnosis of limitations and diffuse lung parenchymal diseases, with advantages such as minimal invasiveness, a high diagnostic positivity rate, and high safety. Due to the strong irritancy of TBLB procedures, patients experience significant discomfort. To improve patient tolerance and provide a better operating environment for endoscopists, TBLB is typically performed under sedation or anesthesia. Complications associated with TBLB include bleeding, respiratory depression, and pneumothorax. Although the overall incidence is low, the mortality rate is high once they occur, which is still a concern. Massive bleeding related to bronchoscopy procedures is defined as acute massive bleeding of the lower respiratory tract caused by bronchoscopy procedures, a single episode of bleeding ≥ 100 ml, either diagnostic or therapeutic. Due to its sudden onset, rapid bleeding, and difficulty in stopping it, it can quickly lead to airway obstruction, causing asphyxiation or even shock and death. A large retrospective study showed that during diagnostic bronchoscopy, the probability of major bleeding from biopsy procedures is the highest, reaching 46.39%, with a mortality rate as high as 10.8%.^[4]^ Therefore, the management of acute major bleeding during TBLB is particularly important, requiring close cooperation and a calm response from anesthesiologists and endoscopy teams.

Sedation for TBLB is usually performed in the bronchoscopy room, which differs from the operating room. In the bronchoscopy room, there is limited space for operation, incomplete ventilation and monitoring equipment required for anesthesia, and medical staff are not familiar with anesthesia work. In case of emergency, it is difficult to cooperate and support the anesthesiologist effectively, and the anesthesia risk is higher than that in the operating room. Additionally, since the anesthesiologist and endoscopist share the airway during surgery, it becomes more difficult to control the airway in case of intra-airway bleeding. Therefore, sedation for bronchoscopy outside the operating room is fraught with risks, and the management of acute bleeding during the procedure presents a significant challenge for anesthesiologists.

In this case, the patient experienced acute massive hemorrhage during TBLB, with bleeding volume instantly exceeding 100 ml, leading to a loss of view in the bronchoscope. The key to the successful resuscitation of this patient lay in immediately adjusting the patient’s position to the left lateral position upon discovering left-lung hemorrhage and performing continuous suction to remove blood from the airway. When the view was lost, the bronchoscope was withdrawn, and tracheal intubation was performed decisively. An 8.0-mm inner-diameter endotracheal tube was inserted into the main airway without performing lung isolation. Bleeding in the airway is more commonly managed with extended insertion of the endotracheal tube into the healthy lung, double-lumen endotracheal tubes, or occlusion of the diseased lung with a blocker. However, these methods are difficult to perform when visualization is lost, especially in cases of massive hemorrhage, and it is challenging to stop bleeding in the affected lung after isolation. Some studies have used two tracheal tubes simultaneously – one placed in the healthy side to maintain ventilation and the other in the affected side for isolation and hemostasis^[5]^ – but this technique is complex and time-consuming. In this emergency, the use of a large-diameter endotracheal tube combined with suction allowed rapid airway clearance and effective oxygenation, enabling the endoscopist to perform targeted hemostasis. Hemostatic drugs were administered intravenously for systemic control. Considering that severe bleeding may require further intervention, a balloon or blocker can be inserted through the tracheal tube for compression or isolation if necessary. In this case, the large inner-diameter intubation proved effective in maintaining ventilation and facilitating airway management, but this observation is derived from a single case and should be interpreted with caution. Its generalizability is limited and requires confirmation through future studies.

In addition to airway management, internal conditions were closely monitored during resuscitation. The bleeding led to impaired pulmonary ventilation, causing hypoxemia, hypercapnia, and subsequent respiratory acidosis. Hypoxia inhibited aerobic metabolism and, combined with hypovolemia-induced metabolic acidosis, required timely correction. After establishing the airway, fluid resuscitation, administration of 100 mL sodium bicarbonate, and transfusion of 2 U of packed red blood cells were initiated, which stabilized the internal milieu and provided time for airway management. Postoperative blood gas analysis confirmed the efficacy of these measures.

The role of tranexamic acid in controlling airway bleeding has received increasing attention in recent years. Both systemic and topical (nebulized) administration have been reported to effectively reduce hemoptysis volume and shorten bleeding duration with a favorable safety profile.^[6–8]^ Randomized and observational studies have demonstrated that nebulized tranexamic acid (typically 500 mg 3 times daily) can safely reduce bleeding and hospital stay duration in hemoptysis patients.^[6,7]^ Recent reviews also highlight its low cost and accessibility, suggesting it as an effective adjunctive measure for airway bleeding.^[8]^

According to the Chinese Expert Consensus on the Prevention and Management of Bronchoscopic Procedure-Related Bleeding, tranexamic acid (500–1000 mg IV or topical) may be used as an adjunctive hemostatic agent, although its onset of action is relatively slow and its hemostatic effect is less pronounced than that of pituitrin or hemocoagulase. In this case, the bleeding was massive and developed rapidly within seconds after biopsy, necessitating immediate airway control and the use of faster-acting hemostatic agents such as pituitrin and hemocoagulase. Once hemostasis was achieved, the patient’s condition stabilized, and therefore tranexamic acid was not administered. Nevertheless, based on current evidence, tranexamic acid remains a valuable adjunct in managing moderate or persistent hemoptysis and should be considered when the clinical situation allows.

Preoperative assessment of bleeding risk and preparation of contingency plans are crucial. Before diagnostic bronchoscopy, a comprehensive evaluation of the patient’s bleeding risk should be conducted, including medical history, comorbidities, cardiopulmonary function, laboratory tests, and imaging studies. Special attention should be paid to the patient’s platelet count, PT, APTT, etc., during biopsy procedures to rule out severe coagulation disorders. The patient’s condition was due to chronic inflammation and a history of atrial fibrillation, routinely taking rivaroxaban for anticoagulation. To prevent intraoperative bleeding, the patient stopped taking rivaroxaban 24 hours before surgery. Since preoperative liver function tests showed mild impairment and prolonged antibiotic use may have reduced endogenous vitamin K synthesis, vitamin K_1_ was administered to support hepatic synthesis of vitamin K-dependent coagulation factors. However, vitamin K_1_ does not antagonize rivaroxaban; its action is limited to vitamin K-dependent factors. Pre‑operative intramuscular Vitamin K_1_ was not administered for urgent reversal of massive intraoperative hemorrhage; instead, it served as pre‑operative optimization. The goal was to correct potential underlying Vitamin K_1_ deficiency and optimally restore the patient’s coagulation substrate reserves prior to surgery, as part of comprehensive pre‑operative risk modification. For this high‑risk patient with pre‑operatively elevated INR, intramuscular Vitamin K_1_ was adopted as a low‑risk, potentially beneficial adjunctive measure to reduce intraoperative bleeding risk, given the unavailability of specific reversal agents. With a half‑life of 24 to 48 hours, pre‑operative dosing enabled peak procoagulant effects to coincide with the surgical procedure. This represented prophylactic administration rather than emergent rescue therapy. Although PT remained slightly elevated, thorough pre-anesthesia preparation – including resuscitation drugs, tracheal tubes of different sizes, high-frequency ventilation, and an anesthesia machine – facilitated successful management of the crisis.

This is a retrospective case. During the episode of intraoperative oxygen desaturation, hemodynamic changes occurred rapidly, and partial real‑time monitoring data were lost. Consequently, the exact nadir values of heart rate and blood pressure were not documented, which limits further quantitative analysis of hemodynamic alterations accompanying hypoxemia.

In summary, this case demonstrates that rapid airway control and multidisciplinary coordination are crucial for successful management of massive bleeding during bronchoscopy. The use of a large-diameter endotracheal tube with suction proved effective and lifesaving in this particular situation by maintaining airway patency and enabling targeted hemostasis. Continuous risk assessment, early identification of high-risk patients, and adherence to expert consensus remain essential for minimizing mortality associated with bronchoscopy-related hemorrhage. It should be emphasized that this airway management approach is based solely on the experience of a single case. Its efficacy cannot be generalized, and it is not universally applicable. Further investigations with larger sample sizes are required to confirm its clinical value.

## Author contributions

**Conceptualization:** Shuxian Ma.

**Writing—review & editing:** Shuxian Ma, Hongmei Lu.

**Data curation:** Sichen Cui.

**Formal analysis:** Sichen Cui.

**Methodology:** Lining Huang.

**Writing—original draft:** Lining Huang, Hongmei Lu.
